# Ethyl 1-benzyl-5-{[(isopropyl­amino)(3-nitro­phen­oxy)methyl­idene]amino}-1*H*-1,2,3-triazole-4-carboxyl­ate

**DOI:** 10.1107/S1600536810046659

**Published:** 2010-11-17

**Authors:** Hong-Mei Wang, Shou-Heng Deng, Xiao-Hua Zeng, Ping Chen, Feng-Jun Cao

**Affiliations:** a Institute of Medicinal Chemistry, Hubei Medical University, Shiyan 442000, People’s Republic of China; b Center of Oncology, People’s Hospital affiliated with Hubei Medical University, Shi Yan 442000, People’s Republic of China

## Abstract

In the title compound, C_22_H_24_N_6_O_5_, the triazole ring is essentially planar with a maximum deviation of 0.005 (2) Å and forms dihedral angles of 79.78 (11) and 86.22 (11)° with the phenyl and benzene rings, respectively. In the crystal, mol­ecules are linked by inter­molecular N—H⋯N, C—H⋯O and C—H⋯π inter­actions into a three-dimensional network.

## Related literature

For the biological activity of 8-aza­guanine derivatives, see: Roblin *et al.* (1945 ▶); Ding *et al.* (2004 ▶); Mitchell *et al.* (1950 ▶); Levine *et al.* (1963 ▶); Montgomery *et al.* (1962 ▶); Yamamoto *et al.* (1967 ▶); Bariana (1971 ▶); Holland *et al.* (1975 ▶). For related structures, see: Chen & Shi (2006 ▶); Ferguson *et al.* (1998 ▶); Li *et al.* (2004 ▶); Maldonado *et al.* (2006 ▶); Wang *et al.* (2006 ▶); Xiao & Shi (2007 ▶); Zeng *et al.* (2006 ▶, 2009 ▶); Zhao, Hu *et al.* (2005 ▶); Zhao, Wang & Ding (2005 ▶); Zhao, Xie *et al.* (2005 ▶).
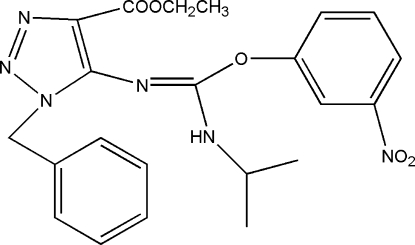


## Experimental

### 

#### Crystal data

C_22_H_24_N_6_O_5_
*M* *_r_* = 452.47
Monoclinic, 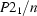

*a* = 11.5019 (7) Å
*b* = 14.5616 (9) Å
*c* = 14.1758 (9) Å
β = 106.384 (1)°
*V* = 2277.8 (2) Å^3^
*Z* = 4
Mo *K*α radiation
μ = 0.10 mm^−1^
*T* = 298 K
0.16 × 0.12 × 0.10 mm

#### Data collection

Bruker SMART CCD area-detector diffractometer
Absorption correction: multi-scan (*SADABS*; Bruker, 2001 ▶) *T*_min_ = 0.985, *T*_max_ = 0.990
16973 measured reflections
5620 independent reflections
4276 reflections with *I* > 2σ(*I*)
*R*_int_ = 0.069

#### Refinement

*R*[*F* ^2^ > 2σ(*F* ^2^)] = 0.067
*wR*(*F* ^2^) = 0.170
*S* = 1.11
5620 reflections
304 parameters
H atoms treated by a mixture of independent and constrained refinement
Δρ_max_ = 0.25 e Å^−3^
Δρ_min_ = −0.19 e Å^−3^

### 

Data collection: *SMART* (Bruker, 2001 ▶); cell refinement: *SAINT* (Bruker, 2001 ▶); data reduction: *SAINT*; program(s) used to solve structure: *SHELXS97* (Sheldrick, 2008 ▶); program(s) used to refine structure: *SHELXL97* (Sheldrick, 2008 ▶); molecular graphics: *PLATON* (Spek, 2009 ▶); software used to prepare material for publication: *SHELXTL* (Sheldrick, 2008 ▶).

## Supplementary Material

Crystal structure: contains datablocks global, I. DOI: 10.1107/S1600536810046659/rz2518sup1.cif

Structure factors: contains datablocks I. DOI: 10.1107/S1600536810046659/rz2518Isup2.hkl

Additional supplementary materials:  crystallographic information; 3D view; checkCIF report

## Figures and Tables

**Table 1:** Hydrogen-bond geometry (Å, °) *Cg*1 and *Cg*2 are the centroids of the triazole and C1–C6 phenyl rings, respectively.

*D*—H⋯*A*	*D*—H	H⋯*A*	*D*⋯*A*	*D*—H⋯*A*
N5—H5*A*⋯N3^i^	0.83 (2)	2.50 (2)	3.230 (2)	148 (2)
C3—H3⋯O1^ii^	0.93	2.42	3.303 (3)	158
C21—H21⋯*Cg*1^ii^	0.93	2.98	3.829 (3)	153
C14—H14⋯*Cg*2^iii^	0.98	2.78	3.625 (2)	145

Symmetry codes: (i) 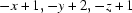; (ii) 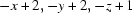; (iii) 
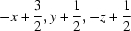
.

**Fig. 1.**
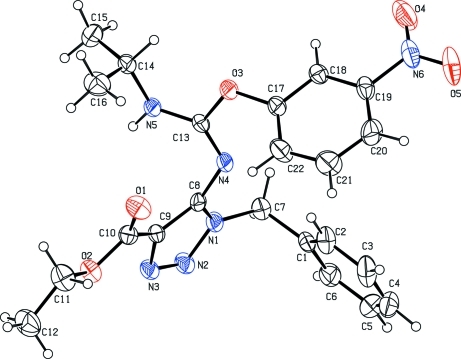
View of the molecule of showing the atom-labeling scheme. Displacement ellipsoids are drawn at 50% probability level. H-atoms are represented by circles of arbitrary size.

## supplementary crystallographic information

### Comment

The derivatives of heterocycles containing 8-azaguanine system, which are well known bioisosteres of guanine, are of great importance because of their remarkable biological properties. Some of these activities include antimicrobial or antifungal activities (Roblin *et al.*, 1945; Ding *et al.*, 2004), encephaloma cell inhibitor activity (Mitchell *et al.*, 1950; Levine *et al.*, 1963), antileukemie activity (Montgomery *et al.*, 1962), hypersusceptibility inhibitor activity and acesodyne activity (Yamamoto *et al.*, 1967; Bariana, 1971; Holland *et al.*, 1975). In recent years, our group has been engaged in the preparation of derivatives of 8-azaguanine *via* aza-Wittig reaction of beta-ethoxycarbonyl iminophosphorane with aromatic isocyanate (Zhao, Xie *et al.*, 2005). As a continuation of our research for new biologically active heterocycles, the title compound was obtained as an intermediate product from β-ethoxycarbonyl iminophosphorane with an alphalic isocyanate, and structurally characterized in this context.

In the title compound (Fig. 1), bond lengths and angles within the triazole ring are in good agreement with those observed for closely related structures (Zhao, Hu *et al.*, 2005; Zhao, Wang & Ding, 2005). As reported for related compounds (Ferguson *et al.*, 1998; Li *et al.*, 2004; Maldonado *et al.*, 2006; Zeng *et al.*, 2006, 2009; Wang *et al.*, 2006; Xiao & Shi, 2007; Chen & Shi, 2006), the triazole ring system is essentially planar, with a maximum displacement of 0.005 (2) Å for atom N3, and forms dihedral angles of 79.78 (11) and 86.22 (11)°  with the phenyl and benzene rings, respectively. In the crystal packing, molecules are linked by intermolecular N—H···N, C—H···O and C—H···π hydrogen bonding interactions (Table 1) in a three-dimensional network.

### Experimental

To a solution of carbodiimide prepared according to Wang *et al.* (2006) in a mixed solvent (CH_2_Cl_2_/CH_3_CN, 1:4 *v*/*v*, 15 ml) was added 3-nitrobenzene (3 mmol), and the reaction mixture was stirred for 6 h. The solvent was removed under reduced pressure and the residue was recrystallized from EtOH to give the title compound in 92% yield (m.p. 437 K). Elemental analysis: calculated for C_22_H_24_N_6_O_5_: C, 58.40; H, 5.35; N, 18.57%. Found: C, 58.08; H, 5.49; N, 18.29%. Crystals suitable for X-ray diffraction study were obtained by recrystallization from EtOH and dichloromethane (1:3 *v*/*v*) at room temperature.

### Refinement

The H atom attached to atom N5 was located in a difference Fourier map and allowed to ride on their parent atom with a restraint of N—H = 0.83 Å and *U*_iso_(H) = 1.2*U*_eq_(N). Other H atoms were placed at calculated positions and treated as riding atoms, with C—H = 0.93–0.97 Å, and *U*_iso_(H) = 1.2*U*_eq_(C) or 1.5*U*_eq_(C) for methyl H atoms.

### Crystal data

**Table:**

C_22_H_24_N_6_O_5_	*F*(000) = 952
*M**_r_* = 452.47	*D*_x_ = 1.319 Mg m^−^^3^
Monoclinic, *P*2_1_/*n*	Mo *K*α radiation, λ = 0.71073 Å
Hall symbol: -P 2yn	Cell parameters from 4925 reflections
*a* = 11.5019 (7) Å	θ = 2.3–27.5°
*b* = 14.5616 (9) Å	µ = 0.10 mm^−^^1^
*c* = 14.1758 (9) Å	*T* = 298 K
β = 106.384 (1)°	Block, colourless
*V* = 2277.8 (2) Å^3^	0.16 × 0.12 × 0.10 mm
*Z* = 4	

### Data collection

**Table:**

Bruker SMART CCD area-detector diffractometer	5620 independent reflections
Radiation source: fine-focus sealed tube	4276 reflections with *I* > 2σ(*I*)
graphite	*R*_int_ = 0.069
φ and ω scans	θ_max_ = 28.3°, θ_min_ = 2.0°
Absorption correction: multi-scan (*SADABS*; Bruker, 2001)	*h* = −15→15
*T*_min_ = 0.985, *T*_max_ = 0.990	*k* = −19→11
16973 measured reflections	*l* = −18→18

### Refinement

**Table:**

Refinement on *F*^2^	Primary atom site location: structure-invariant direct methods
Least-squares matrix: full	Secondary atom site location: difference Fourier map
*R*[*F*^2^ > 2σ(*F*^2^)] = 0.067	Hydrogen site location: inferred from neighbouring sites
*wR*(*F*^2^) = 0.170	H atoms treated by a mixture of independent and constrained refinement
*S* = 1.11	*w* = 1/[σ^2^(*F*_o_^2^) + (0.0702*P*)^2^ + 0.3769*P*] where *P* = (*F*_o_^2^ + 2*F*_c_^2^)/3
5620 reflections	(Δ/σ)_max_ = 0.001
304 parameters	Δρ_max_ = 0.25 e Å^−^^3^
0 restraints	Δρ_min_ = −0.19 e Å^−^^3^

### Special details

**Table:**

Geometry. All e.s.d.'s (except the e.s.d. in the dihedral angle between two l.s. planes) are estimated using the full covariance matrix. The cell e.s.d.'s are taken into account individually in the estimation of e.s.d.'s in distances, angles and torsion angles; correlations between e.s.d.'s in cell parameters are only used when they are defined by crystal symmetry. An approximate (isotropic) treatment of cell e.s.d.'s is used for estimating e.s.d.'s involving l.s. planes.
Refinement. Refinement of *F*^2^ against ALL reflections. The weighted *R*-factor *wR* and goodness of fit *S* are based on *F*^2^, conventional *R*-factors *R* are based on *F*, with *F* set to zero for negative *F*^2^. The threshold expression of *F*^2^ > σ(*F*^2^) is used only for calculating *R*-factors(gt) *etc*. and is not relevant to the choice of reflections for refinement. *R*-factors based on *F*^2^ are statistically about twice as large as those based on *F*, and *R*- factors based on ALL data will be even larger.

### Fractional atomic coordinates and isotropic or equivalent isotropic displacement parameters (Å^2^)

**Table:**

	*x*	*y*	*z*	*U*_iso_*/*U*_eq_	
C1	0.83494 (16)	0.73129 (13)	0.46761 (13)	0.0443 (4)	
C2	0.92546 (19)	0.79033 (16)	0.45953 (18)	0.0607 (6)	
H2	0.9053	0.8448	0.4245	0.073*	
C3	1.0448 (2)	0.7693 (2)	0.5027 (2)	0.0778 (8)	
H3	1.1051	0.8096	0.4969	0.093*	
C4	1.0756 (2)	0.6893 (3)	0.5545 (2)	0.0880 (10)	
H4	1.1565	0.6761	0.5855	0.106*	
C5	0.9865 (3)	0.6285 (2)	0.56061 (19)	0.0858 (9)	
H5	1.0073	0.5731	0.5936	0.103*	
C6	0.8662 (2)	0.64966 (17)	0.51778 (17)	0.0634 (6)	
H6	0.8061	0.6088	0.5227	0.076*	
C7	0.70362 (17)	0.75548 (14)	0.42216 (15)	0.0496 (5)	
H7A	0.6892	0.7643	0.3520	0.060*	
H7B	0.6531	0.7050	0.4318	0.060*	
C8	0.67486 (14)	0.92697 (13)	0.43699 (12)	0.0386 (4)	
C9	0.64906 (14)	0.97754 (13)	0.51172 (12)	0.0374 (4)	
C10	0.65826 (15)	1.07626 (14)	0.52721 (13)	0.0428 (4)	
C11	0.6438 (2)	1.19830 (17)	0.63312 (18)	0.0666 (6)	
H11A	0.5876	1.2347	0.5832	0.080*	
H11B	0.7256	1.2178	0.6369	0.080*	
C12	0.6194 (3)	1.2108 (2)	0.7301 (2)	0.0843 (8)	
H12A	0.5398	1.1884	0.7264	0.126*	
H12B	0.6244	1.2749	0.7468	0.126*	
H12C	0.6784	1.1773	0.7795	0.126*	
C13	0.65271 (15)	1.00782 (13)	0.29254 (12)	0.0405 (4)	
C14	0.48968 (17)	1.11872 (14)	0.21120 (13)	0.0473 (5)	
H14	0.5274	1.1187	0.1572	0.057*	
C15	0.35540 (19)	1.09763 (18)	0.16922 (17)	0.0655 (6)	
H15A	0.3457	1.0371	0.1413	0.098*	
H15B	0.3194	1.1417	0.1191	0.098*	
H15C	0.3164	1.1008	0.2207	0.098*	
C16	0.5115 (3)	1.21091 (17)	0.26153 (18)	0.0714 (7)	
H16A	0.4759	1.2116	0.3152	0.107*	
H16B	0.4754	1.2582	0.2153	0.107*	
H16C	0.5971	1.2216	0.2861	0.107*	
C17	0.82682 (16)	1.01845 (14)	0.23484 (13)	0.0435 (4)	
C18	0.85824 (16)	0.97580 (13)	0.15946 (13)	0.0432 (4)	
H18	0.7997	0.9553	0.1038	0.052*	
C19	0.98042 (18)	0.96439 (14)	0.16946 (15)	0.0496 (5)	
C20	1.06905 (19)	0.99366 (19)	0.25005 (19)	0.0682 (7)	
H20	1.1505	0.9845	0.2549	0.082*	
C21	1.0339 (2)	1.0371 (2)	0.32389 (19)	0.0785 (8)	
H21	1.0926	1.0580	0.3792	0.094*	
C22	0.9133 (2)	1.04989 (19)	0.31693 (16)	0.0652 (6)	
H22	0.8903	1.0794	0.3670	0.078*	
N1	0.66986 (13)	0.83894 (11)	0.46539 (11)	0.0430 (4)	
N2	0.64102 (15)	0.83418 (12)	0.55231 (12)	0.0498 (4)	
N3	0.62788 (13)	0.91804 (12)	0.57907 (11)	0.0447 (4)	
N4	0.70987 (13)	0.94745 (12)	0.35461 (11)	0.0455 (4)	
N5	0.54469 (13)	1.04458 (12)	0.28047 (11)	0.0439 (4)	
H5A	0.5090 (19)	1.0317 (14)	0.3220 (16)	0.053*	
N6	1.0155 (2)	0.91968 (15)	0.08856 (18)	0.0698 (6)	
O1	0.69213 (14)	1.12893 (11)	0.47476 (11)	0.0593 (4)	
O2	0.62878 (12)	1.10169 (9)	0.60849 (10)	0.0500 (3)	
O3	0.70407 (11)	1.03569 (10)	0.22136 (9)	0.0528 (4)	
O4	0.9375 (2)	0.89508 (16)	0.01765 (16)	0.1013 (7)	
O5	1.1227 (2)	0.91031 (17)	0.09688 (19)	0.1151 (8)	

### Atomic displacement parameters (Å^2^)

**Table:**

	*U*^11^	*U*^22^	*U*^33^	*U*^12^	*U*^13^	*U*^23^
C1	0.0471 (10)	0.0477 (11)	0.0431 (9)	0.0052 (8)	0.0210 (8)	−0.0076 (8)
C2	0.0551 (12)	0.0505 (13)	0.0834 (15)	0.0011 (10)	0.0306 (11)	−0.0126 (11)
C3	0.0489 (12)	0.0810 (19)	0.108 (2)	−0.0053 (12)	0.0295 (13)	−0.0411 (17)
C4	0.0548 (14)	0.125 (3)	0.0771 (17)	0.0327 (17)	0.0069 (12)	−0.0258 (18)
C5	0.088 (2)	0.101 (2)	0.0679 (16)	0.0427 (18)	0.0212 (14)	0.0184 (15)
C6	0.0686 (14)	0.0688 (16)	0.0606 (13)	0.0095 (12)	0.0308 (11)	0.0101 (11)
C7	0.0496 (10)	0.0482 (12)	0.0541 (11)	0.0008 (9)	0.0196 (9)	−0.0052 (9)
C8	0.0280 (7)	0.0500 (11)	0.0404 (9)	0.0051 (7)	0.0140 (6)	0.0050 (8)
C9	0.0305 (7)	0.0485 (11)	0.0368 (8)	−0.0005 (7)	0.0152 (6)	0.0024 (8)
C10	0.0324 (8)	0.0556 (12)	0.0417 (9)	−0.0059 (8)	0.0126 (7)	−0.0004 (9)
C11	0.0732 (15)	0.0536 (14)	0.0767 (15)	−0.0157 (11)	0.0271 (12)	−0.0179 (12)
C12	0.098 (2)	0.0805 (19)	0.0757 (17)	0.0005 (15)	0.0265 (14)	−0.0294 (15)
C13	0.0398 (9)	0.0514 (11)	0.0351 (8)	0.0012 (8)	0.0184 (7)	0.0005 (8)
C14	0.0497 (10)	0.0560 (12)	0.0424 (9)	0.0108 (9)	0.0232 (8)	0.0099 (9)
C15	0.0512 (12)	0.0804 (17)	0.0640 (13)	0.0159 (11)	0.0150 (10)	0.0107 (12)
C16	0.0987 (18)	0.0556 (14)	0.0640 (14)	0.0020 (13)	0.0295 (13)	0.0087 (12)
C17	0.0412 (9)	0.0518 (11)	0.0438 (9)	0.0010 (8)	0.0222 (7)	0.0068 (8)
C18	0.0454 (9)	0.0456 (11)	0.0429 (9)	−0.0027 (8)	0.0197 (8)	0.0027 (8)
C19	0.0514 (11)	0.0474 (12)	0.0607 (12)	0.0034 (9)	0.0334 (9)	0.0061 (9)
C20	0.0402 (10)	0.0885 (18)	0.0812 (16)	−0.0018 (11)	0.0255 (11)	0.0057 (14)
C21	0.0529 (13)	0.112 (2)	0.0677 (15)	−0.0206 (13)	0.0130 (11)	−0.0204 (15)
C22	0.0605 (13)	0.0871 (18)	0.0546 (12)	−0.0113 (12)	0.0270 (10)	−0.0182 (12)
N1	0.0407 (8)	0.0483 (10)	0.0453 (8)	0.0047 (7)	0.0209 (6)	0.0040 (7)
N2	0.0539 (9)	0.0551 (11)	0.0489 (9)	0.0017 (8)	0.0280 (7)	0.0075 (8)
N3	0.0471 (8)	0.0511 (10)	0.0423 (8)	0.0000 (7)	0.0228 (7)	0.0038 (7)
N4	0.0443 (8)	0.0578 (10)	0.0419 (8)	0.0127 (7)	0.0242 (6)	0.0081 (7)
N5	0.0410 (8)	0.0572 (10)	0.0401 (8)	0.0081 (7)	0.0224 (6)	0.0109 (7)
N6	0.0799 (14)	0.0630 (13)	0.0853 (15)	0.0147 (11)	0.0538 (12)	0.0007 (11)
O1	0.0667 (9)	0.0590 (9)	0.0594 (8)	−0.0195 (7)	0.0296 (7)	0.0017 (7)
O2	0.0562 (8)	0.0491 (8)	0.0505 (7)	−0.0073 (6)	0.0244 (6)	−0.0071 (6)
O3	0.0456 (7)	0.0770 (10)	0.0447 (7)	0.0127 (7)	0.0271 (6)	0.0163 (7)
O4	0.1143 (17)	0.1171 (18)	0.0855 (13)	0.0095 (13)	0.0492 (13)	−0.0371 (13)
O5	0.0924 (14)	0.129 (2)	0.156 (2)	0.0250 (13)	0.0866 (15)	−0.0155 (16)

### Geometric parameters (Å, °)

**Table:**

C1—C2	1.380 (3)	C13—N5	1.319 (2)
C1—C6	1.380 (3)	C13—O3	1.367 (2)
C1—C7	1.507 (3)	C14—N5	1.476 (2)
C2—C3	1.371 (3)	C14—C16	1.508 (3)
C2—H2	0.9300	C14—C15	1.522 (3)
C3—C4	1.370 (4)	C14—H14	0.9800
C3—H3	0.9300	C15—H15A	0.9600
C4—C5	1.375 (5)	C15—H15B	0.9600
C4—H4	0.9300	C15—H15C	0.9600
C5—C6	1.379 (4)	C16—H16A	0.9600
C5—H5	0.9300	C16—H16B	0.9600
C6—H6	0.9300	C16—H16C	0.9600
C7—N1	1.462 (2)	C17—C18	1.370 (3)
C7—H7A	0.9700	C17—C22	1.378 (3)
C7—H7B	0.9700	C17—O3	1.393 (2)
C8—N1	1.350 (2)	C18—C19	1.382 (3)
C8—N4	1.371 (2)	C18—H18	0.9300
C8—C9	1.389 (2)	C19—C20	1.367 (3)
C9—N3	1.361 (2)	C19—N6	1.471 (3)
C9—C10	1.454 (3)	C20—C21	1.377 (3)
C10—O1	1.207 (2)	C20—H20	0.9300
C10—O2	1.342 (2)	C21—C22	1.376 (3)
C11—O2	1.448 (3)	C21—H21	0.9300
C11—C12	1.489 (3)	C22—H22	0.9300
C11—H11A	0.9700	N1—N2	1.365 (2)
C11—H11B	0.9700	N2—N3	1.300 (2)
C12—H12A	0.9600	N5—H5A	0.83 (2)
C12—H12B	0.9600	N6—O4	1.198 (3)
C12—H12C	0.9600	N6—O5	1.212 (3)
C13—N4	1.286 (2)		
			
C2—C1—C6	119.1 (2)	N5—C14—C15	108.05 (16)
C2—C1—C7	120.50 (19)	C16—C14—C15	112.33 (19)
C6—C1—C7	120.38 (19)	N5—C14—H14	108.6
C3—C2—C1	120.5 (2)	C16—C14—H14	108.6
C3—C2—H2	119.7	C15—C14—H14	108.6
C1—C2—H2	119.7	C14—C15—H15A	109.5
C4—C3—C2	120.3 (3)	C14—C15—H15B	109.5
C4—C3—H3	119.9	H15A—C15—H15B	109.5
C2—C3—H3	119.9	C14—C15—H15C	109.5
C3—C4—C5	119.8 (2)	H15A—C15—H15C	109.5
C3—C4—H4	120.1	H15B—C15—H15C	109.5
C5—C4—H4	120.1	C14—C16—H16A	109.5
C4—C5—C6	120.1 (3)	C14—C16—H16B	109.5
C4—C5—H5	120.0	H16A—C16—H16B	109.5
C6—C5—H5	120.0	C14—C16—H16C	109.5
C5—C6—C1	120.2 (2)	H16A—C16—H16C	109.5
C5—C6—H6	119.9	H16B—C16—H16C	109.5
C1—C6—H6	119.9	C18—C17—C22	121.54 (18)
N1—C7—C1	111.57 (16)	C18—C17—O3	117.06 (16)
N1—C7—H7A	109.3	C22—C17—O3	121.18 (17)
C1—C7—H7A	109.3	C17—C18—C19	117.32 (18)
N1—C7—H7B	109.3	C17—C18—H18	121.3
C1—C7—H7B	109.3	C19—C18—H18	121.3
H7A—C7—H7B	108.0	C20—C19—C18	123.04 (19)
N1—C8—N4	120.53 (16)	C20—C19—N6	119.04 (19)
N1—C8—C9	103.88 (14)	C18—C19—N6	117.92 (19)
N4—C8—C9	135.35 (18)	C19—C20—C21	117.96 (19)
N3—C9—C8	108.45 (16)	C19—C20—H20	121.0
N3—C9—C10	122.91 (15)	C21—C20—H20	121.0
C8—C9—C10	128.12 (16)	C22—C21—C20	120.9 (2)
O1—C10—O2	123.88 (19)	C22—C21—H21	119.5
O1—C10—C9	123.91 (17)	C20—C21—H21	119.5
O2—C10—C9	112.17 (15)	C21—C22—C17	119.2 (2)
O2—C11—C12	107.5 (2)	C21—C22—H22	120.4
O2—C11—H11A	110.2	C17—C22—H22	120.4
C12—C11—H11A	110.2	C8—N1—N2	111.03 (15)
O2—C11—H11B	110.2	C8—N1—C7	128.74 (15)
C12—C11—H11B	110.2	N2—N1—C7	119.81 (16)
H11A—C11—H11B	108.5	N3—N2—N1	107.07 (15)
C11—C12—H12A	109.5	N2—N3—C9	109.55 (14)
C11—C12—H12B	109.5	C13—N4—C8	121.01 (14)
H12A—C12—H12B	109.5	C13—N5—C14	126.48 (15)
C11—C12—H12C	109.5	C13—N5—H5A	117.0 (15)
H12A—C12—H12C	109.5	C14—N5—H5A	115.6 (15)
H12B—C12—H12C	109.5	O4—N6—O5	123.3 (2)
N4—C13—N5	130.36 (16)	O4—N6—C19	118.8 (2)
N4—C13—O3	117.65 (15)	O5—N6—C19	118.0 (2)
N5—C13—O3	111.79 (15)	C10—O2—C11	115.68 (16)
N5—C14—C16	110.70 (16)	C13—O3—C17	118.53 (14)
			
C6—C1—C2—C3	−1.6 (3)	C9—C8—N1—N2	0.76 (18)
C7—C1—C2—C3	178.52 (19)	N4—C8—N1—C7	3.4 (3)
C1—C2—C3—C4	0.0 (4)	C9—C8—N1—C7	−171.70 (16)
C2—C3—C4—C5	2.0 (4)	C1—C7—N1—C8	89.8 (2)
C3—C4—C5—C6	−2.4 (4)	C1—C7—N1—N2	−82.1 (2)
C4—C5—C6—C1	0.8 (4)	C8—N1—N2—N3	−0.05 (19)
C2—C1—C6—C5	1.1 (3)	C7—N1—N2—N3	173.18 (15)
C7—C1—C6—C5	−179.0 (2)	N1—N2—N3—C9	−0.72 (19)
C2—C1—C7—N1	−62.2 (2)	C8—C9—N3—N2	1.21 (19)
C6—C1—C7—N1	117.9 (2)	C10—C9—N3—N2	−171.13 (16)
N1—C8—C9—N3	−1.17 (17)	N5—C13—N4—C8	−16.3 (3)
N4—C8—C9—N3	−175.21 (18)	O3—C13—N4—C8	169.37 (17)
N1—C8—C9—C10	170.66 (17)	N1—C8—N4—C13	136.47 (19)
N4—C8—C9—C10	−3.4 (3)	C9—C8—N4—C13	−50.3 (3)
N3—C9—C10—O1	170.10 (17)	N4—C13—N5—C14	175.4 (2)
C8—C9—C10—O1	−0.7 (3)	O3—C13—N5—C14	−10.0 (3)
N3—C9—C10—O2	−7.7 (2)	C16—C14—N5—C13	−94.9 (2)
C8—C9—C10—O2	−178.49 (15)	C15—C14—N5—C13	141.7 (2)
C22—C17—C18—C19	0.8 (3)	C20—C19—N6—O4	−179.2 (2)
O3—C17—C18—C19	175.51 (16)	C18—C19—N6—O4	0.0 (3)
C17—C18—C19—C20	−0.1 (3)	C20—C19—N6—O5	0.2 (3)
C17—C18—C19—N6	−179.29 (18)	C18—C19—N6—O5	179.4 (2)
C18—C19—C20—C21	−0.5 (4)	O1—C10—O2—C11	−2.0 (3)
N6—C19—C20—C21	178.6 (2)	C9—C10—O2—C11	175.81 (16)
C19—C20—C21—C22	0.5 (4)	C12—C11—O2—C10	−174.08 (18)
C20—C21—C22—C17	0.2 (4)	N4—C13—O3—C17	−17.6 (3)
C18—C17—C22—C21	−0.8 (4)	N5—C13—O3—C17	166.98 (17)
O3—C17—C22—C21	−175.3 (2)	C18—C17—O3—C13	128.60 (19)
N4—C8—N1—N2	175.89 (14)	C22—C17—O3—C13	−56.7 (3)

### Hydrogen-bond geometry (Å, °)

**Table:**

Cg1 and Cg2 are the centroids of the triazole and C1–C6 phenyl rings, respectively.

**Table:**

*D*—H···*A*	*D*—H	H···*A*	*D*···*A*	*D*—H···*A*
N5—H5A···N3^i^	0.83 (2)	2.50 (2)	3.230 (2)	148 (2)
C3—H3···O1^ii^	0.93	2.42	3.303 (3)	158
C21—H21···Cg1^ii^	0.93	2.98	3.829 (3)	153
C14—H14···Cg2^iii^	0.98	2.78	3.625 (2)	145

Symmetry codes: (i) −*x*+1, −*y*+2, −*z*+1; (ii) −*x*+2, −*y*+2, −*z*+1; (iii) −*x*+3/2, *y*+1/2, −*z*+1/2.
